# The Impact of Psychopharmacology on Contemporary Psychiatry

**DOI:** 10.1177/070674371405900801

**Published:** 2014-08

**Authors:** Ross J Baldessarini

**Affiliations:** 1 Professor of Psychiatry and in Neuroscience, Department of Psychiatry, Harvard Medical School, Boston, Massachusetts; Founding Director, International Bipolar & Psychotic Disorder Research Consortium, Mailman Research Center, McLean Hospital, Boston, Massachusetts.

**Keywords:** drug development, psychiatric education, practice, theory, psychopharmacology

Modern clinical psychopharmacology can be dated from the introduction of lithium carbonate to treat mania by John Cade in Australia in 1949 or from the introduction of chlorpromazine as the first synthetic drug found to be effective in both mania and psychotic disorders in Paris in the early 1950s. Soon thereafter, the mood-elevating effects of the monoamine oxidase inhibitor, iproniazid, and the antidepressant effects of imipramine were reported. By 1960, haloperidol, the first butyrophenone antipsychotic, the first so-called atypical antipsychotic, clozapine, and the benzodiazepines also were introduced.^1^ That is, at least one agent from each major class of currently employed psychotropic drugs was known by the end of the 1950s. These new treatments brought about fundamental changes in the treatment of many major psychiatric disorders of unknown cause—notably, mania, depression, acute and chronic psychotic disorders, including schizophrenia, as well as severe anxiety disorders. These changes can fairly be considered revolutionary. Moreover, their impact extended far beyond improvements in treatment, and included fundamental changes in the conceptualization of most psychiatric disorders, in their diagnosis and categorization, on models for research into the nature of psychiatric illnesses, on psychiatric education, on methods and standards for experimental therapeutics, and on the organization of modern psychiatry as a clinical and academic medical specialty.

In the first 2 decades of their introduction into psychiatric therapeutics, there was an intense struggle among the previous generation of psychiatrists who had been captivated by the psychodynamic and psychoanalytic tradition initiated by Sigmund Freud and his followers in the early 1900s. A common early assertion was that the new drugs might modify symptoms and limit pain and suffering, but left undone much of what was required to bring about major and sustained changes in behaviour and thinking. Nevertheless, a new generation of more medically or biologically oriented psychiatrists came to dominate psychiatry internationally, and to replace their more psychologically minded colleagues in positions of influence, including most university chairs of psychiatry.

This historical perspective is particularly timely for this issue of *The Canadian Journal Psychiatry*, aimed at a critical assessment of where clinical psychopharmacology and its impact on psychiatry now stand. Such sensitive questions arise as to whether the pharmacotherapeutic approach may have been overdone, with widespread degradation of standards for patient assessment and comprehensive care, as well as deeply affecting psychiatric education and the organization and functioning of psychiatric institutions and systems of care delivery.^2^,^3^ As noted by Dr David M Gardner^4^ and Dr Gustavo H Vázquez,^5^ there has appeared, in recent decades, a growing international inclination toward increasingly brief and routinized clinical encounters, with an emphasis on rapid but superficial diagnostic categorization and initiation of almost exclusively medicinal treatments. Even if such clinical practices were adequate, Dr Gardner^4^ emphasizes that they require extensive training, experience, knowledge, and judgment to be used effectively and safely. The argument can be made that heavy reliance on medicinal treatments with less emphasis on psychological approaches, and on symptom checklists rather than on thoughtful understanding of each patient has brought about fundamental changes in the theory and practice of modern psychiatry. These changes involve shifting the balance of tension between what has been labelled brainlessness versus mindlessness in psychiatry, in the biomedical direction.^6^,^7^ Such changes, in turn, are consistent with compelling efforts in recent decades to manage (limit) the costs of medical care of all types. Questions to be considered include whether this shift may be antithetical to comprehensive, thoughtful, and individualized care of people with psychiatric illness, and whether it provides an adequate model for psychiatric training.

Another profound effect of the introduction of effective and reasonably safe and tolerated medicinal treatments for psychiatric illnesses has been to reframe the tasks and possibilities for academic psychiatry and psychiatric research in a more biomedical perspective. This shift in interest was greatly stimulated by major technical and experimental advances that gave birth to the new field of neuroscience since the 1960s, culminating in recent explosive advances in structural and functional brain imaging, behavioural and neurogenetics, and molecular neuroscience.^8^ The changes in therapeutic practice as well as in research orientation marked a return to the 19th-century tradition of neuromedically oriented and descriptive psychiatry—a tradition that became neglected in the early-to-mid 20th century.^3^ A more biomedical approach is attractive, but may remain premature, and surely is an incomplete basis for understanding of most mental illnesses. As increasingly technically sophisticated and detailed information is developed in such fields as neuroimaging and neurogenetics, we are repeatedly reminded that almost all major mental disorders remain fundamentally idiopathic. Most lack not only known etiologies but also even a coherent pathophysiology. This fundamental truism limits efforts to develop a biomedically oriented psychiatry beyond the empirical application of psychotropics and detection of occasional coarse neurological disorders. Nevertheless, clinical and research efforts to develop a more biomedical psychiatry are appropriate and of great, but largely potential or even hypothetical, value. Indeed, the history of a series of movements in biology and medicine brought to address psychiatric disorders during the past 2 centuries is marked by time-limited enthusiasm, limited progress, and moving on to the next conceptual fashion.^9^ A point that is directly relevant to the present discussion of psychopharmacology is that the lack of a pathophysiology, let alone an etiology, for most psychiatric illnesses makes rational progress in therapeutics extremely difficult and highly risky from both a scientific and business perspective.

A fundamental aspect of the great leaps forward of psychopharmacology in the unprecedently innovative era of the 1950s is that nearly all of the discoveries of novel treatments and therapeutic theories rested not on rational prediction or laboratory experimentation arising from a secure pathological or pathophysiological basis, but largely on chance observations with immediate clinical implications—that is, the process of serendipity. Examples include the surprising clinical effects of lithium carbonate when used mainly for its putative anti-gout activity; observations, initially by surgeons and anesthesiologists, that chlorpromazine was not merely another sedative; unexpected mood changes in tuberculosis sanatoria on introduction of the *N*-isopropyl analog of isoniazid; surprising mood changes with imipramine, which looks chemically rather like another tricyclic antipsychotic; the counter-surprise that clozapine, though chemically rather imipraminelike, was not mood-elevating; and there are many others.^1^ A remarkable observation is that serendipity has not yet been replaced by modern neurobiology or advances in industrial or academic chemistry and neuropharmacology. Again, this conclusion follows from the lack of a tissue pathology or a plausible pathophysiology for most mental disorders.

A consequence of these circumstances is that there has been remarkably little fundamental innovation in psychopharmacologic therapeutics for psychiatry since the early 1960s. Most recently introduced psychotropics are modelled on chemical or pharmacodynamic similarities to earlier predecessors. This process has provided a viable business model and has led to patentable and often highly profitable new drug products, but very little that is fundamentally new or improved. In addition, psychotropic markets are saturating, patent protection is ending, and drug development pipelines are drying up. Indeed, there is a growing sense among pharmacological investigators and the pharmaceutical industry that we are stuck. In turn, a growing number of corporations are shifting investment and resources away from the central nervous system to apparently more tractable clinical problems that have indeed witnessed some striking and fundamental innovations in recent years.^10^ A consequence of the lack of innovation in psychotropic treatments, as emphasized by Dr Gardner,^4^ is that psychiatry is obliged to redouble efforts to make the best use of what we have while hoping for the next therapeutic breakthrough—whether guided by scientific theory or again through serendipity.

A further effect of the discovery of the several new classes of psychotropics in and following the 1950s is that a new kind of biological theorizing became dominant in academic psychiatry—one that I have termed pharmacocentric.^1^,^9^ The basic idea is that, as the science of drug action (pharmacodynamics) has made initial small advances, it has been irresistibly tempting to argue that the opposite of the drug action may be a clue to pathophysiology. Among other examples, this kind of thinking led to the dopamine-excess theory of psychotic disorders and mania based on the antidopaminergic actions of most antipsychoticantimanic drugs, to various monoamine deficiency hypotheses concerning depression and some anxiety disorders based on speculations about the norepinephrineor serotonin-potentiating actions of most antidepressants. Although such theorizing stimulated a generation of clever experimentation, findings of research aimed at testing them at the clinical level has remained inconsistent and unconvincing. This outcome should not be any more surprising than, for example, expecting to discover the pneumococcus from detailed knowledge of the molecular pharmacology of willow bark and its antipyretic salicylates. An extension of such speculations sometimes extends into clinical practice, as diagnoses or rationales for particular treatments are presented to patients couched in concepts arising from pharmacodynamics but representing little more than neuromythology. Again, the fundamental fact is that the disorders considered to lie within the province of psychiatry remain idiopathic.

The changes outlined above have had additional, fundamental effects on the theory and practice of psychiatry. One is that the shift away from 19th-century interests in a neurobiology of mental illness was also associated with a decline in interest in classic descriptive psychiatry and in psychopathology. This loss of interest largely continues, even in European centres where the tradition developed.^11^ It has also been accompanied by some peculiar developments in both psychiatric diagnosis and clinical practice. Regarding nosology, a former handful of credible psychiatric diagnoses^12^ has grown into a massive collection of hundreds of putative disorders to be found in standard international diagnostic manuals.^13^–^15^ Most of these are largely imperfectly defined and minimally investigated by traditional epidemiologic methods, continue to lack a coherent biology, and sometimes prove to be limited in clinical and research utility in the face of often complex or atypical clinical presentations. Examples include the highly unstable group of acute psychoses, most of which evolve into other disorders on follow-up,^16^,^17^ and the nearly incoherent group of major depressive disorders.^18^,^19^ At the level of clinical practice, there is a strong temptation to simplify and generalize. To an antipsychotic, antidepressant, or mood-stabilizing hammer, many conditions look like nails. And yet, efforts to differentiate and optimize drug responses among clinical conditions or clinically defined types of patients remain primitive or ignored. Pressures to maintain broad, relatively nonspecific, markets for various types of psychotropics have been very high, as noted by Dr Vázquez.^5^ It has been tempting for both the pharmaceutical industry and psychiatric clinicians to ascribe great weight to findings of statistically significant improvements in randomized, placebo-controlled trials. Indeed, the early years of modern psychopharmacology were at the forefront of development of current standard methods of design and analysis of controlled, clinical therapeutic trials. The problem is that most findings arise (quite appropriately) from trials designed to gain regulatory approval and to pursue the aims of a marketing plan, rather than to inform and refine rational clinical practice. Ironically, massive treasures of information about clinical subtypes of patients who did especially well or poorly with a given treatment, or tolerated it especially well or poorly, remain in computer banks held as proprietary information by pharmaceutical manufacturers, and left minimally evaluated by clinical investigators of all kinds. A far more sophisticated body of information is needed to inform and guide sound clinical practice. This information reasonably can include information on clinical subtypes and a more refined set of expectations of what a given treatment can reasonably be expected to do.

Dr Vázquez^5^ identifies additional factors that limit the ability of contemporary therapeutics research to contribute to a more sophisticated, specific, and predictable application of the available medicines that Dr Gardner^4^ challenges us to use more wisely. These include the tendency to generalize from averaged findings obtained with highly selected, often clinically unrepresentative, patient-subjects in therapeutic trials, and to make averages of averages in the currently enthusiastic application of data-pooling by the methods of meta-analysis. This kind of therapeutics research can usually identify useless or grossly intolerable compounds, but can hardly be expected to produce refined guidance for such basic clinical questions as which drug to start with and in what doses and for how long and for whom . . . and then what? In addition to excessive generalization (for example, all forms of depression respond well and safely to antidepressants; antipsychotics are adequate treatment for all manifestations of schizophrenia), there is a tendency to overvalue or exaggerate the therapeutic efficacy of most psychotropics. In turn, such exaggerated expectations may arise from overvaluing probability values in comparisons of active drugs and placebos, rather than to attend to more relevant effect sizes (difference in drug, compared with placebo, response divided by variance of measurement).

Regarding such outcome measures, there is both good and bad news. Reassuring news includes findings of a recent, ambitious, and scholarly comparison of effect sizes of psychotropics to medicines employed in general medicine, which found relatively favourable results for many psychotropics.^20^ Not so good are clinically apparent tendencies to expect more of antipsychotics, antidepressants, mood stabilizers, or anxiolytics than they may deliver clinically with individual patients. Drug superiorities to placebo controls are typically in the range of 30% to 50%, often with impressive *P* values (which can be engineered to assure success of even a marginally effective treatment provided that the number of subjects is high^21^). For example, antidepressants may average 30% to 40% higher rates of response than a randomly assigned placebo treatment, but such numbers can be misleading. Rarely do trial outcomes represent full clinical, symptomatic, or functional recovery. Instead, they typically involve changes in standardized rating scale scores (which may or may not be adequate surrogates for clinical assessment), usually aim for improvements as low as 50%, are carried out only for perhaps 6 or 8 weeks, and involve highly selected subjects who may not adequately represent clinically encountered patients with nominally similar diagnoses. Rather, similar averaged outcomes within any class of psychotropics are virtually inevitable as drugs would not be marketed if not superior to placebo in at least 2 (of sometimes numerous) trials. Indeed, it has proved difficult to demonstrate substantial, credible, and clinically meaningful differences between specific drugs within a given class in terms of efficacy and tolerability, whether they be antidepressants, anxiolytics, antipsychotics, or proposed mood stabilizers.^22^–^24^ Overall, despite their limitations, available averaged outcomes for most types of clinically employed psychotropics are generally favourable. Nevertheless, such evidence is far from being a sound basis on which to assume that the work of modern psychiatric therapeutics is simply to pick the right drug and an approximately appropriate dose for a given patient.

In addition, evidence for long-term effectiveness of most types of psychotropics in providing sustained benefits and protection from recurrences of psychiatric illnesses remains particularly limited and often based on ambiguous research methods.^1^ These include the potentially biasing selection of patients who respond, short term, to a given drug-product (whose manufacturer typically sponsors the trial) to continue (relatively briefly in comparison to the natural history of recurrence patterns) into aftercare that often involves randomized discontinuation to a placebo—that is, removing an apparently effective treatment, often with incomplete recovery from an acute illness. Such trial designs can add drug-discontinuation stress to factors associated with relapses or recurrences of illness, and so inflate apparent drug–placebo differences.^25^,^26^ Drug discontinuation can not only confound interpretation of long-term treatment trials but also sometimes have potentially dangerous, adverse effects on clinical treatment. Such risks are particularly evident in pregnancy, when many women and their physicians are more concerned with usually hypothetical or rare teratogenic effects than with common and major adverse effects on maternal health, and their unknown effects on the developing fetus.^27^,^28^

A striking consequence arises from the evidently widely accepted belief that psychotropics routinely provide major clinical benefits and that a solution to most clinical problems can be found with the right drug or combination of drugs at the right doses. Such beliefs encourage what can be termed an allopathic compulsion to pursue pharmacological treatments relentlessly, uncritically, often rather thoughtlessly, and potentially dangerously. Moreover, given substantial risks of failure and high rates of partial or temporary symptomatic improvements with standard psychotropic treatments given in monotherapy, there is a growing temptation to try imaginative combinations or agents, higher than recommended doses, or to add unconventional treatments that lack regulatory approval for psychiatric applications. Most such practices appear to be responses to patient and clinician frustrations with lack of substantial clinical improvement. Even though some such efforts are often understandable, almost always, they lack specific scientific testing for added effectiveness with acceptable safety.^29^–^32^

Given the appreciable limitations of modern psychotropic medicines to solve the complex human problems represented by most cases of psychiatric illness, it seems especially ironic that much of what psychiatry learned during the past 2 centuries—including efforts to develop descriptive nosologies, and psychopathological as well as psychodynamic understanding of people with psychiatric illness—appears to have become devalued in the competition with seemingly simple, effective, supposedly even sufficient, and certainly cost-effective, pharmacologically based treatments. This view of psychiatry is strongly encouraged by currently pervasive interest in savings of costs, time, and effort in this era of managed care. As has occurred with many previous movements in psychiatry (descriptive, psychopathological, neuromedical, psychodynamic, community psychiatry, and others), psychopharmacology is currently overvalued, and at long-term risk of being devalued or even abandoned, too. As noted by Dr Vázquez,^5^ an increasingly ominous trend in psychiatric clinical practice and in training programs, is the difficulty to engage in curiosity. The decline of curiosity has been encouraged by increasing pressures to produce more units of clinical product per hour, to save as much time and cost as possible, and to avoid asking questions that may seem to complicate understanding or care of a patient.

In summary, modern psychopharmacology has brought clinical benefits that have truly revolutionized modern psychiatry. It also has had a profound impact on nosology and theories of psychiatric illnesses, on hypotheses for psychiatric research, as well as on the training of mental health clinicians and the organization of contemporary mental health services. Nevertheless, it has led to some notable, unintended, adverse consequences. These appear to arise from overestimating and overvaluing the effectiveness and tolerability of psychotropic treatments, sometimes with relentless pursuit of increasingly complex, aggressive, and nonrational treatment regimens whose value and safety are untested. Consequences of the domination of modern psychiatric therapeutics by drug treatments include wide disparities in the quality of patient assessment, treatment, and follow-up care, surely encouraged by cost-containment efforts throughout clinical medicine. There is a particularly lamentable threat to the tradition of thoughtfulness, thoroughness, and curiosity that has evolved during the past 2 centuries of progress in psychiatry.
